# Temperature-Dependent Viscosity Analysis of Powell–Eyring Fluid Model during a Roll-over Web Coating Process

**DOI:** 10.3390/polym16121723

**Published:** 2024-06-17

**Authors:** Fateh Ali, Srikantha Narasimhamurthy, Soniya Hegde, Muhammad Usman

**Affiliations:** 1 College of Mathematics and System Sciences, Xinjiang University, Urumqi 830046, China; 2 Department of Mathematics, M.S. Ramaiah Institute of Technology, Visvesvaraya Technological University, Bengaluru 560054, Karnataka, India; 3 School of Mathematics & Statistics, Xi’an Jiaotong University, Xi’an 710049, China

**Keywords:** rollover web coating, variable viscosity, analytic solutions, lubrication approximation theory, response surface method, perturbation

## Abstract

The roll coating method is of considerable significance in several industries, as it is applied practically in the production of paint, the manufacturing of PVC-coated cloth, and the plastic industry. The current study theoretically and computationally analyses the Powell–Eyring fluids with variable viscosity during the non-isothermal roll-over web phenomenon. Based on the lubrication approximation theory (LAT), the problem was formulated. The system of partial differential equations (PDEs) obtained from the mathematical modeling was further simplified to a set of ordinary differential equations (ODEs) using suitable transformations. A regular perturbation method was implemented to obtain the solution in terms of velocity, pressure gradient, pressure, and flow rate per unit width. This study also captures important engineering characteristics such as coating thickness, Nusselt number, shear stress, roll/sheet separating force, and roll-transmitted power to the fluid. Along with a comparison between the present work and published work, both graphical and tabular representations wer made to study the effects of various factors. It was observed that the velocity profile is the decreasing function of non-Newtonian and Reynold viscosity parameters. In addition, the response surface methodology (RSM) was employed to investigate the sensitivity of the shear stress and the Nusselt number.

## 1. Introduction

The process of applying a substance onto the surface of an object is known as coating. This practice not only embellishes the object but also shields it, enhancing its efficiency, quality, and overall characteristics. Coating solid surfaces with fluid films is a widespread technique in industries. Its applications span various sectors including acrylics, fabrics, electronics, catalogues, metalwork, paper production, magnetic tape recording, printing for magazines and books, X-ray films, wallpapers, and more. Different methods have been developed for coating, with reverse and forward roll coating being among the commonly employed techniques. 

Roll coating is a process through which a uniform film of liquid is deposited on a moving sheet. Theoretical investigations of the roll coating process delve into understanding the fundamental principles governing the application of coatings onto surfaces through rollers. This exploration aims to uncover the underlying mechanisms influencing key parameters such as coating thickness, uniformity, and overall efficiency. By examining factors such as fluid dynamics, material properties, and process conditions, researchers can optimize roll coating techniques to achieve desired outcomes in various industrial applications. These investigations are essential for improving product quality, enhancing production efficiency, and developing innovative solutions for diverse coating challenges.

Studies often focus on elucidating the impact of various parameters such as viscosity, speed, non-Newtonian behavior, pressure, and flow dynamics on the coating process. Greener and Middleman [1] pioneered the initial theoretical examination of roll coating for both Newtonian and non-Newtonian fluids by considering LAT. The effect of the operating parameters on coating thickness and pressure distribution is studied using analytical solutions. Hintermaier and White [2] analyzed the water flow between the two rollers using the lubrication model, and the solutions they derived closely matched their experimental findings. Benkreira et al. [3,4,5] delved into the examination of coating flows for Newtonian fluids as well as several non-Newtonian fluid models, employing both theoretical analyses and experimental investigations. Coyle et al. [6] investigated the fluid dynamics of reverse roll coating, revealing flow instabilities like ribbing and cascades. It explores the importance of the dynamic wetting line in controlling flow behavior. Manzoor et al. [7] present a study investigating the theoretical analysis of roll-over thin layer formation employing LAT (Lubrication Approximation Theory). Their findings unveil the influence of fluid properties on both coating quality and efficiency. The study on roll-over-web coating of micropolar-Casson fluid is investigated by Abbas and Khaliq [8], who employed lubrication theory to analyze velocity, microrotation, and pressure gradient with numerical computations, revealing the effects of viscoplastic parameter and coupling number on engineering variables. The research emphasizes the control mechanisms provided by these parameters for exit sheet thickness, power input, and separating force in the coating process. In light of this, several investigations have explored the utilization of reverse roll coating with various fluid types and are cited in [9,10,11,12].

Despite the practical versatility of non-Newtonian fluids, the Navier–Stokes theory faces limitations in capturing their performance intricacies due to complex rheological properties. In response, several paradigms have emerged to provide insights into the distinct nature of non-Newtonian fluids. One particularly significant model in this realm is the Powell–Eyring fluid model. Eyring and Powell [13] introduced the Eyring–Powell model (PE model) in 1944 as a non-Newtonian fluid model, offering a comprehensive approach to understanding both the plasticity and viscosity features of the fluid. There are two key benefits associated with the Eyring–Powell model. Firstly, its development is grounded in a kinetic liquid molecular theory of gases rather than an empirical relationship. Secondly, the Eyring–Powell liquid undergoes a transition to a Newtonian fluid at both high and low shear rates. Khan et al. [14] investigated the magnetohydrodynamic flow behavior and heat conduction in wire coating with liquid polymers in a porous medium using an Eyring–Powell fluid model. Their study incorporates the effects of joule heating and temperature-dependent viscosity, with results validated through Homotopy Analytical and BVPH2 techniques. A mathematical paradigm defining the coating system of the wire in the presence of Eyring–Powell fluid under a magnetic field and Joule heating effect is investigated by Aljohani et al. [15]. Recently, Fateh et al. [16] discussed the rollover web coating process for the Sisko fluid model using LAT theoretically. The regular perturbation method has been implemented to obtain solutions for velocity, pressure gradient, pressure, and flow rate per unit width. Further, the study provided insights into important engineering characteristics such as coating thickness, Nusselt number, shear stress, roll/sheet separating force, and roll-transmitted power to the fluid. Graphical and tabular representations have been made to compare the work with published work, demonstrating the effects of various factors. The LMA-TNN technique is utilized to solve the designed model. Subsequently, multiple researchers investigated their work, specifically centering on Eyring–Powell fluid, and it is referenced in [17,18,19,20,21,22].

Viscosity stands out as a paramount thermophysical property of fluids, prompting numerous researchers to investigate flows with viscosity as a function of temperature. In scenarios involving significant temperature variations, the fluid’s viscosity becomes highly sensitive. Therefore, it becomes imperative to adopt a model that incorporates viscosity as a function of temperature to avoid inaccuracies in the heat transfer rate coefficient. Also, achieving optimal heat transfer efficiency is a key objective in numerous applications where the variable viscosity model becomes pivotal. The impact of two viscosity models, Reynolds and Vogel’s, on the wire/cable coating process, was analyzed by Bhukta et al. [23], and the findings were compared with those obtained using a constant viscosity model. Srikantha and Hegde [24] conducted a study to investigate how fluctuating thermophysical properties affect the behavior of Sisko fluid during the coating process when subjected to a magnetic field. The research led to the conclusion that an increase in the variable viscosity parameter results in an enhanced velocity, while the temperature distribution undergoes a reduction. Examining this perspective, a variety of studies were carried out to investigate flow patterns by employing the variable viscosity concept (refer to [25,26,27,28]).

On the flip side, practical execution of the analysis places a strong emphasis on optimizing the obtained results. Therefore, it is essential to conduct statistical analysis to assess the significance of selected key factors in enhancing the model’s efficiency. Employing optimization methodology to minimize or maximize objective functions is also a critical aspect. In light of this, several researchers incorporated surface response methodology into their investigations (see [29,30,31,32]).

Examining the structure of the literature mentioned above, it becomes evident that:

- Numerous studies have tackled the process of roll coating involving different non-Newtonian fluids with constant thermophysical properties. Nevertheless, there exists a gap in research that explicitly focuses on the effects of change in viscosity concerning with temperature, particularly in the case of Eyring–Powell fluid as a non-Newtonian fluid.
- Most of the studies do not capture the important engineering characteristics such as coating thickness, Nusselt number, shear stress, roll/sheet separating force, and roll transmitted power to the fluid.
- Studies that center the concurrent optimization of chosen key parameters to maximize heat transfer rate and minimize shear stress rate are scarce.

In view of the highlighted aspects from the literature review, the primary focus of the current work revolves around:

- Constructing an Eyring–Powell fluid model for rollover coating process using Reynold’s model—a variable viscosity model.
- Investigating the outcome of temperature-dependent viscosity parameter in a rollover web coating problem.
- Examining the consequence of interested pertinent parameters on the engineering key factors like coating thickness, stress rate, rate of heat transfer coefficient, roll/sheet separating force, and roll transmitted power to the fluid.
- Identifying the limitations that achieve the dual goals of maximizing the rate of heat transfer and minimizing stress rate simultaneously through the application of optimization methods.

## 2. Governing Equations and the Problem Formulation

The momentum, mass, and energy equations in the absence of body forces are as follows:

(1) ∇⋅V=0,

(2) $$\rho \frac{DV}{Dt}=\nabla \tau -\nabla p,$$

(3) $$\rho C_{p}\left(\frac{DT}{Dt}\right)=k\nabla^{2}T+\tau \cdot \nabla V,$$

where V→ velocity field, p → pressure, k → thermal conductivity, T → temperature profile, ρ → constant fluid density, $C_{p}$ → specific heat, and τ → stress tensor.

The material derivative is given as

(4) $$\frac{D(\cdot )}{Dt}=\frac{\partial (\cdot )}{\partial t}+(V\cdot \nabla )(\cdot ),$$

**Figure 1:**
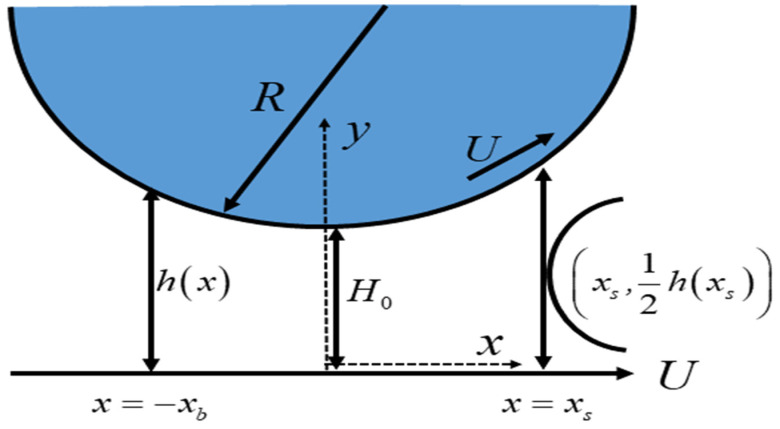
Geometry for rollover web coating process.

The following assumptions are made:

⮚ A steady, incompressible, and non-isothermal flow of Eyring–Powell fluid with temperature-dependent viscosity is considered for the rollover web coating procedure.
⮚ The radius of the roll is taken as R, which rotates counterclockwise with an angular velocity represented by ω, resulting in the linear velocity U=ωR.
⮚ As the roll starts rotating, the web or sheet starts moving towards a positive x-direction with a constant velocity U.
⮚ The web and roll have a small separation region called the nip area and the length of that gap is $H_{0}$, as depicted in Figure 1.
⮚ Assuming $H_{0}/R\ll 1$, the flow is locally regarded as a flow between two parallel plates.

When working with complex physical systems, scientists and engineers often simplify mathematical models to create useful approximations. This process aims to provide a better understanding of the physical system by focusing on key aspects such as fluxes, forces, velocities, and other relevant components. The above assumptions lead us to apply lubrication approximation theory (LAT). The LAT suggests that the most critical dynamic events primarily occur within the nip region, offering a foundation for detailed analysis.

For laminar, incompressible, and steady two-dimensional flow, the velocity profile is as follows:

(5) $$V=\left[u\left(x,\;y\right),\;v\left(x,\;y\right)\right]$$

### 2.1. Rheological Model

This study examines the rheological properties as described by the viscoelastic Powell–Eyring model. The mathematical representation of the PE model is given by the following equations:

(6) $$\tau =\mu A_{1}+\frac{1}{\bar{B}}\frac{\sinh^{-1}\left(\frac{\sqrt{tr\left(A_{1}^{2}\right)}}{2\bar{C}}\right)}{\frac{\sqrt{tr\left(A_{1}^{2}\right)}}{2}}A_{1},$$

where $A_{1}$ is the first Rivilin Ericksen tensor, which is given as:

(7) $$A_{1}={\left(\nabla V\right)}^{T}+\nabla V$$

From Equations (1)–(6), the components form can be written as follows:

(8) $$\frac{\partial u}{\partial x}+\frac{\partial v}{\partial y}=0,$$

(9) $$\rho \left(u\frac{\partial u}{\partial x}+v\frac{\partial u}{\partial y}\right)=-\frac{\partial p}{\partial x}+\frac{\partial \tau_{xx}}{\partial x}+\frac{\partial \tau_{xy}}{\partial y},$$

(10) $$\rho \left(u\frac{\partial v}{\partial x}+v\frac{\partial v}{\partial y}\right)=-\frac{\partial p}{\partial y}+\frac{\partial \tau_{yx}}{\partial x}+\frac{\partial \tau_{yy}}{\partial y},$$

(11) $$\tau_{xy}=\left(\mu +\frac{1}{\bar{B}\bar{C}}\right)\frac{\partial u}{\partial y}-\frac{1}{6\bar{B}{\bar{C}}^{3}}{\left(\frac{\partial u}{\partial y}\right)}^{3},$$

(12) $$\rho C_{p}\left(u\frac{\partial T}{\partial x}+v\frac{\partial T}{\partial y}\right)=k\left[\frac{\partial^{2}T}{\partial x^{2}}+\frac{\partial^{2}T}{\partial y^{2}}\right]+\tau_{xx}\frac{\partial u}{\partial y}+\tau_{xy}\left(\frac{\partial u}{\partial y}+\frac{\partial v}{\partial x}\right)+\tau_{yy}\frac{\partial v}{\partial y}.$$

### 2.2. Non-Dimensionalities

The following non-dimensional parameters are used to non-dimensionalize the system:

(13) $$\begin{array}{l} x^{*}=\frac{x}{\sqrt{2RH_{0}}},\;u^{*}=\frac{u}{U},\;y^{*}=\frac{y}{H_{0}},\;v^{*}=\frac{v}{U}\sqrt{\frac{R_{0}}{H_{0}}},\;p^{*}=\sqrt{\frac{H_{0}}{2R}}\frac{pH_{0}}{aU},\\ Q^{*}=\frac{Q}{2UH_{0}},\tau_{xy}^{*}=\frac{\tau_{xy}H_{0}}{aU}\;,\;h^{*}(x^{*})=\frac{h\left(x\right)}{H_{0}}=1+x^{2},T^{*}=\frac{T-T_{0}}{T_{1}-T_{0}}, \end{array}$$

Using Equation (13), the governing equations in their non-dimensional form are as follows:

(14) $$\frac{\partial u}{\partial x}+\frac{\partial v}{\partial y}=0,$$

(15) εReu∂u∂x+v∂u∂y=−∂p∂x+δ∂τxx∂x+∂τxy∂x,

(16) ε2Reu∂u∂x+v∂u∂y=−∂p∂y+δ∂τyy∂y+δ2∂τxy∂x,

(17) $$\frac{\partial^{2}T}{\partial y^{2}}+Br\tau_{xy}\frac{\partial u}{\partial y}=0,$$

(18) $$\tau_{xy}=\left(\mu +\bar{N}\right)\frac{\partial u}{\partial y}-\frac{1}{3}\bar{N}\beta {\left(\frac{\partial u}{\partial y}\right)}^{3},$$

(19) $$\mu \frac{\partial^{2}u}{\partial y^{2}}+\frac{\partial \mu }{\partial y}\frac{\partial u}{\partial y}+\bar{N}\left(1-\frac{1}{3}\beta {\left(\frac{\partial u}{\partial y}\right)}^{2}\right)\frac{\partial^{2}u}{\partial y^{2}}=\frac{dp}{dx},$$

(20) $$\frac{\partial^{2}T}{\partial y^{2}}+Br\left(\mu +\bar{N}\right){\left(\frac{\partial u}{\partial y}\right)}^{2}-\frac{1}{3}\bar{N}\beta Br{\left(\frac{\partial u}{\partial y}\right)}^{4}=0.$$

The boundary conditions in the dimensionless form:

(21) $$\left\{\begin{array}{c} u=1,\;\mathrm{on}\;y=h\left(x\right),\\ u=1,\tau_{xy}=0,\;\mathrm{on}\;y=0. \end{array}\right.$$

Under the conditions described, $h\left(x\right)$ denotes the variable height between the sheet and the roll.

### 2.3. Viscosity Model (Reynolds Model)

The Reynolds model [33] gives the expression for the viscosity as a function of temperature as follows:

(22) $$\mu =e^{-MT},$$

where $M=\alpha \left(T_{1}-T_{0}\right)$, (α is any constant). Let M=We m, where We and m is a small perturbation and viscosity variation parameters, respectively. Using Taylor expansion, Equation (22) reduces to

(23) $$\mu =1-We\;m\;T,\;\frac{d\mu }{dy}=-We\;m\frac{dT}{dy}\;.$$

Considering the aforementioned circumstances, the equations governing motion and temperature are defined by:

(24) $$\left(1-WemT\right)\frac{d^{2}u}{dy^{2}}-Wem\frac{dT}{dy}\frac{du}{dy}+WeN\frac{d^{2}u}{dy^{2}}-\frac{WeN\beta }{3}{\left(\frac{du}{dy}\right)}^{2}\frac{d^{2}u}{dy^{2}}=\frac{dp}{dx}$$

(25) $$\frac{d^{2}T}{dy^{2}}+Br\left(1-WemT\right){\left(\frac{du}{dy}\right)}^{2}+BrWeN{\left(\frac{du}{dy}\right)}^{2}-\frac{BrWeN\beta }{3}{\left(\frac{du}{dy}\right)}^{4}=0$$

The perturbation expansions for momentum and energy equations in this case are as follows:

(26) $$\begin{array}{l} u=u_{0}+Weu_{1}+We^{2}u_{2},\;T=T_{0}+WeT_{1}+We^{2}T_{2},\\ \frac{dp}{dx}=We^{2}\frac{dp_{2}}{dx}+We\frac{dp_{1}}{dx}+\frac{dp_{0}}{dx},\;\lambda =We^{2}\lambda_{2}+We\lambda_{1}+\lambda_{0}. \end{array}$$

where $\lambda =Q/UH_{0}$ is the dimensionless flow rate parameter derived from the quantity volumetric flow rate $Q=\int_{0}^{h(x)}udy$(see [1]).

By expanding the equations and matching the coefficients of $We^{0}$, $We^{1}$ and so forth, we derive a series of boundary value problems. In the subsequent subsection, we focus solely on the zero and first-order problems.

The zeroth order $\left(We^{0}\right)$ BVP and its analytical solution for the velocity profile are as follows:

(27) $$\frac{d^{2}u_{0}}{dy^{2}}=\frac{dp_{0}}{dx}$$

(28) $$u_{0}=1,\;\mathrm{at}\;y=1,\;u_{0}=1,\;\mathrm{at}\;y=h(x)$$

(29) $$u_{0}=\frac{\left(\frac{d}{dx}p_{0}(x)\right)y^{2}}{2}-\frac{h\left(\frac{d}{dx}p_{0}(x)\right)y}{2}+1$$

The flow rate in non-dimensional form is

(30) $$\lambda_{0}=\int_{0}^{h(x)}u_{0}dy$$

Substituting $u_{0}$ from Equation (29) in Equation (30), the pressure distribution is obtained as

(31) $$\frac{dp_{0}}{dx}=12\frac{h(x)-\lambda_{0}}{{\left(h(x)\right)}^{3}}$$

Using Equation (31) in Equation (29), the final form of velocity is obtained as,

(32) $$u_{0}(x,y)=1+6\left(\frac{h-\lambda_{0}}{h^{3}}\right)\left(y^{2}-hy\right)$$

The velocity will be zero at the point of separation $\left(x_{s},\frac{1}{2}h\left(x_{s}\right)\right)$, where the fluid divides equally to coat both the roll and the sheet. Hence, from Equation (32), we can write

(33) $$x_{s}=\sqrt{3\lambda_{0}-1}$$

On integrating Equation (31), it takes the following form

(34) $$p_{0}=12\int_{x_{s}}^{x}\frac{h(x)-\lambda_{0}}{h{\left(x\right)}^{3}}$$

(35) $$p_{0}=\frac{\left(6-\left(9/2\right)\lambda_{0}\right)x}{1+(x^{2}/2)}-\frac{3\lambda_{0}x}{{\left(1+\left(x^{2}/2\right)\right)}^{2}}+\left(\frac{12}{\sqrt{2}}-\frac{9\lambda_{0}}{\sqrt{2}}\right)tan^{-1}\frac{x}{\sqrt{2}}+\frac{6\pi }{\sqrt{2}}\left(1-\frac{3\lambda_{0}}{4}\right),$$

The most basic dynamic model of the separation zone is predicated on the premise that the film divides at the precise location where $u_{0}=0$ and $p_{0}=0$, specifically, those of velocity, pressure, and temperature are satisfied.

Incorporating Equation (33) into Equation (35) yields a transcendental equation in $\lambda_{0}$, which is solved using the Newton–Raphson method. For the zeroth order, the flow rate is calculated as 1.3015, accurate to four decimal places, with a residual error of −0.00046. It is important to note that the coating, as mentioned earlier, thickness is attained precisely at the separation point $x_{0}=2.4102$. Notably, these zero-order results closely resemble those found by Sofou and Mitsoulis [34].

The first-order BVP and its analytical solution for the velocity profile are as follows:

(36) $$\frac{d^{2}u_{1}}{dy^{2}}-mT_{0}\frac{d^{2}u_{0}}{dy^{2}}-m\frac{dT_{0}}{dy}\frac{du_{0}}{dy}+N\frac{d^{2}u_{0}}{dy^{2}}-\frac{N\beta }{3}{\left(\frac{du_{0}}{dy}\right)}^{2}\frac{d^{2}u_{0}}{dy^{2}}=\frac{dp_{1}}{dx}$$

(37) $$u_{1}=0,\;\mathrm{at}\;y=0,\;u_{0}=0,\;\mathrm{at}\;y=h(x)$$

(38) $$u_{1}\left(x,y\right)=\frac{y}{11520x^{2}+23040}\left(160\left(\begin{array}{l} \left(-\frac{GBrm}{5}+N\beta \left(h^{2}-2hy+2y^{2}\right)\right)\left(x^{2}+2\right){\left(\frac{dp_{0}}{dx}\right)}^{3}\\ +\left(\left(12h+48y\right)m-36Nx^{2}-72N\right)\frac{dp_{0}}{dx}\\ +36\frac{dp_{1}}{dx}x^{2}+72\frac{dp_{1}}{dx} \end{array}\right)\left(h-y\right)\right)$$

where G and $\frac{dp_{1}}{dx}$ are given as below:

(39) $$G=2\left(\begin{array}{l} \frac{5y^{4}}{2}+\left(-3x^{2}+h-6\right)y^{3}+\left(h^{2}+\left(-\frac{9}{4}-\frac{9x^{2}}{8}\right)h+\frac{45{\left(x^{2}+2\right)}^{2}}{32}\right)y^{2}\\ +\left(h^{3}+\left(-\frac{9}{4}-\frac{9x^{2}}{8}\right)h^{2}+\frac{15{\left(x^{2}+2\right)}^{2}h}{32}-\frac{5{\left(x^{2}+2\right)}^{3}}{16}\right)y\\ +\left(h^{2}+\left(-\frac{5x^{2}}{8}-\frac{5}{4}\right)h+\frac{5{\left(x^{2}+2\right)}^{2}}{32}\right)\left(-\frac{x^{2}}{2}+h-1\right)h \end{array}\right)$$

(40) $$\frac{dp_{1}}{dx}=\frac{24}{35{\left(x^{2}+2\right)}^{7}}\left(\begin{array}{l} -30Brmx^{10}+180Br\lambda_{0}mx^{8}+70Nx^{10}-35mx^{10}-360Br\lambda_{0}^{2}mx^{6}-\\ 300Brmx^{8}-140N\lambda_{0}x^{8}+70\lambda_{0}mx^{8}+240Br\lambda_{0}^{3}mx^{4}+1440Br\lambda_{0}mx^{6}+\\ 700Nx^{8}-140\lambda_{1}x^{8}-350mx^{8}-2160Br\lambda_{0}^{2}mx^{4}-1200Brmx^{6}-\\ 1120N\lambda_{0}x^{6}-672N\beta x^{6}+560\lambda_{0}mx^{6}+960Br\lambda_{0}^{3}mx^{2}+4320Br\lambda_{0}mx^{4}+\\ 4032N\lambda_{0}\beta x^{4}+2800Nx^{6}-1120\lambda_{1}x^{6}-1400mx^{6}-4320Br\lambda_{0}^{2}mx^{2}-\\ 2400Brmx^{4}-8064N\lambda_{0}^{2}\beta x^{2}-3360N\lambda_{0}x^{4}-4032N\beta x^{4}+1680\lambda_{0}mx^{4}+\\ 960Br\lambda_{0}^{3}m+5760Br\lambda_{0}mx^{2}+5376N\lambda_{0}^{3}\beta +16128N\lambda_{0}\beta x^{2}+5600Nx^{4}-\\ 3360\lambda_{1}x^{4}-2800mx^{4}-2880Br\lambda_{0}^{2}m-2400Brmx^{2}-16128N\lambda_{0}^{2}\beta -\\ 4480N\lambda_{0}x^{2}-8064N\beta x^{2}+2240\lambda_{0}mx^{2}+2880Br\lambda_{0}m+16128N\lambda_{0}\beta +\\ 5600Nx^{2}-4480\lambda_{1}x^{2}-2800mx^{2}-960Brm-2240N\lambda_{0}-5376N\beta +\\ 1120\lambda_{0}m+2240N-2240\lambda_{1}-1120m \end{array}\right)$$

The corresponding dimensionless flow rate is

(41) $$\lambda_{1}=\int_{0}^{h(x)}u_{1}dy.$$

The solution for the first order will be obtained using a procedure akin to that of the zeroth order.

Now, the zeroth $\left(We^{0}\right)$ and the first $\left(We^{1}\right)$ order BVP along with its analytical solutions for the temperature profile are as below:

(42) $$\frac{d^{2}T_{0}}{dy^{2}}+Br{\left(\frac{du_{0}}{dy}\right)}^{2}=0$$

(43) $$T_{0}=0\;\mathrm{at}\;y=0,\;T_{0}=1\;\mathrm{at}\;y=h(x)$$

(44) $$T_{0}(x,y)=\frac{\left(384+\left(x^{4}+\left(-4y+4\right)x^{2}+8y^{2}-8y+4\right)Br\left(x^{2}-2y+2\right)\left(x^{2}+2\right){\left(\frac{dp_{0}}{dx}\right)}^{2}\right)y}{192x^{2}+384}$$

(45) $$\frac{d^{2}T_{1}}{dy^{2}}+2Br\frac{du_{0}}{dy}\frac{du_{1}}{dy}-BrmT_{0}{\left(\frac{du_{0}}{dy}\right)}^{2}+BrN{\left(\frac{du_{0}}{dy}\right)}^{2}-\frac{BrN\beta }{3}{\left(\frac{du_{0}}{dy}\right)}^{4}=0$$

(46) $$T_{1}=0\mathrm{at}\;y=0,\;T_{1}=0\;\mathrm{at}\;y=h(x)$$

On substituting zeroth and first-order solutions in Equation (26), the velocity, pressure gradient, and temperature profiles solution can be found. Using these, all other interesting engineering quantities such as coating thickness, roll separation force, power input, Nusselt number, and shear rate can be derived.

## 3. Machinal Quantities of Interest 

### 3.1. Coating Thickness

It is worth mentioning that λ has a correlation with the coating thickness H, since

(47) Q=2UH

Hence, the coating thickness in a dimensionless form is as follows:

(48) $$\frac{H}{H_{0}}=\frac{\lambda }{2}$$

### 3.2. Roll Separation Force (F)

The non-dimensional F is achieved through

(49) $$F=\frac{\bar{F}H_{0}}{\mu (T)URW}=\int_{-\infty }^{x_{s}}p\left(x\right)dx$$

where $\bar{F}$ and F denotes the dimensional and non-dimensional forms of the roll separation force.

### 3.3. Power Input

The power transferred to the fluid from the roll is obtained through integration.

(50) $$p_{w}=\frac{P^{*}}{\mu (T)WU^{2}}=\int_{-\infty }^{x_{s}}\tau_{xy}\left(x,1\right)dx$$

Here, $p_{w}$ and $\tau_{xy}$ signify the non-dimensional power and component form of share stress as specified in the above section.

### 3.4. Nusselt Number

The relation for Nusselt Nu is defined as

(51) $$Nu={\left|\frac{\partial T}{\partial y}\right|}_{y=h}$$

### 3.5. Shear Rate

The expression for shear stress is as follows

(52) $$\tau_{xy}=\left(\mu (T)+\bar{N}\right)\frac{\partial u}{\partial y}-\frac{1}{3}\bar{N}\beta {\left(\frac{\partial u}{\partial y}\right)}^{3}$$

## 4. Results and Discussion

The primary objective of this section is to explain the physical aspects of the emerging parameters on the distributions of velocity, pressure gradient, pressure profile, temperature, and other related engineering quantities. This will be accomplished by taking into consideration the flow of PE fluid with temperature-dependent viscosity during the rollover coating process. The numerical results for the volumetric flow rate, the exit coating thickness, the roll separation force, and the power input for the interested parameters are presented in Table 1, Table 2 and Table 3.

**Figure 2:**
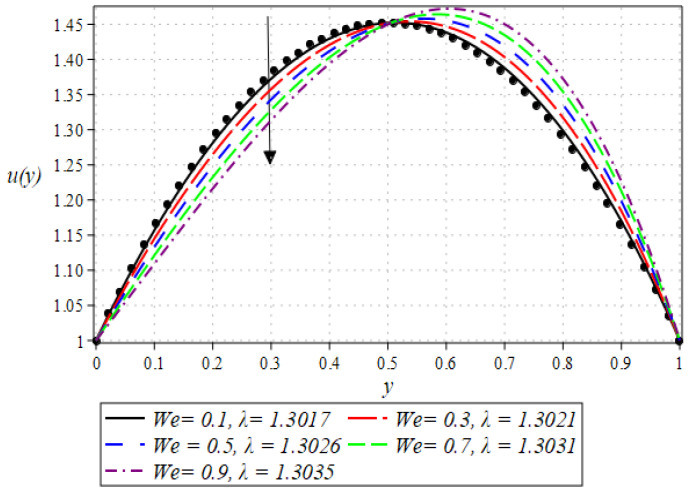
Impact of We on velocity profile.

**Figure 3:**
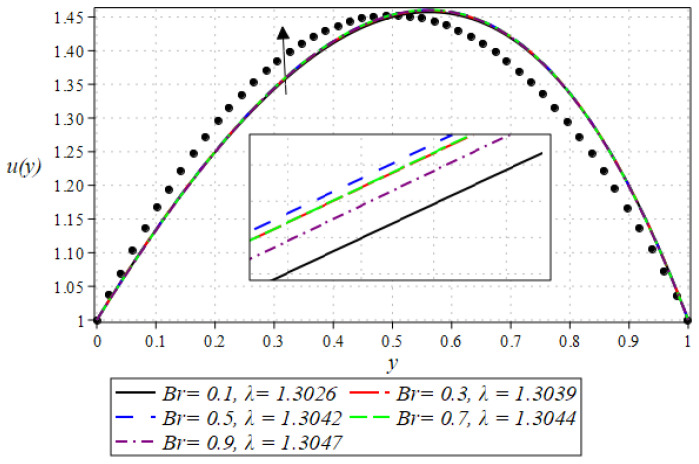
Impact of Br on velocity profile.

**Figure 4:**
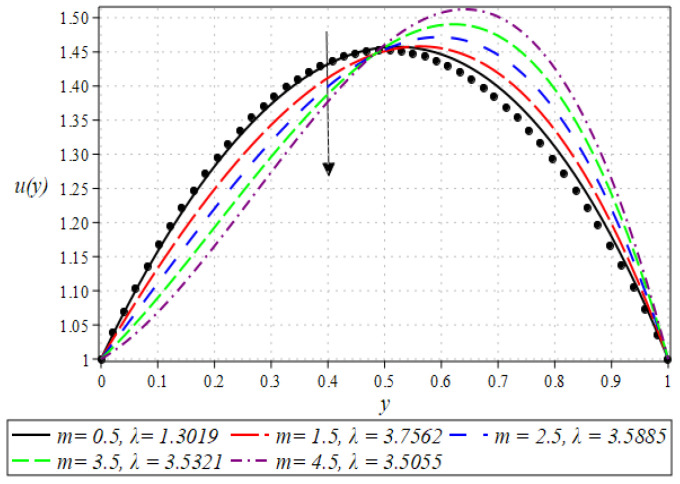
Impact of m on velocity profile.

**Figure 5:**
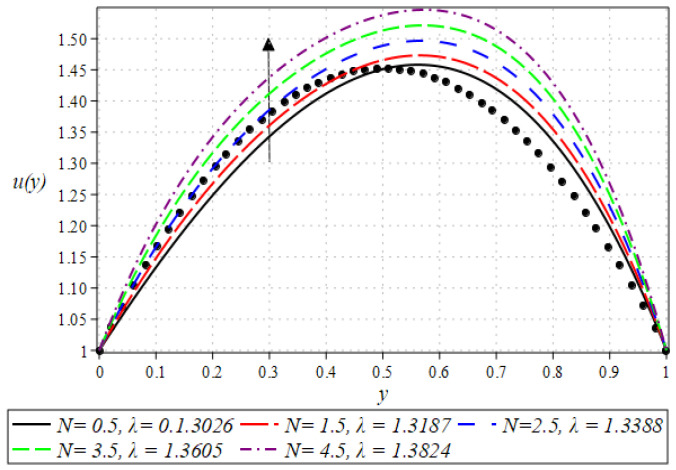
Impact of N on velocity profile.

The outcome for the dimensionless velocity profiles against y for involved parameters during the rollover coating procedure is projected in Figure 2, Figure 3, Figure 4 and Figure 5. The impact of the Weissenberg number on velocity is shown in Figure 2. It has been observed that on increasing the value of We from 0.1 to 0.9, there is a noticeable reduction in the peak velocity within the fluid flow. The We characterizes the ratio of the elastic force to the viscous force in the fluid. At higher We values, the fluid exhibits more pronounced elastic behavior, which tends to resist deformation and slows down the flow. This resistance manifests as a reduced velocity throughout the fluid layer. The impact of the Brinkman number Br can be observed in Figure 3. It is clear from the figure that on increasing the value of Br, the fluid velocity increases. Higher Br values indicate greater heat generation due to viscous effects, which in turn reduces the fluid’s viscosity. Lower viscosity leads to less resistance to flow, thereby increasing the fluid velocity. The next aspect that is shown in Figure 4 is the effect that alterations in the Reynolds viscosity index m. From this plot, it is observed that, increasing the value of m, the velocity of the fluid first decreases and starts increasing after a certain point that is y=0.5. Physically, it seems true, because an increase in the value of m means diminishing absolute viscosity of the fluid and as a result, the transfer of heat predicts the melting effects on the fluid, which rises the fluid velocity. Figure 5 shows the effect of material parameter N on the velocity profile. The velocity of the fluid is increased by increasing the magnitude of parameter N. Next, we discuss plots of the pressure gradient and pressure for emerging parameters.

**Figure 6:**
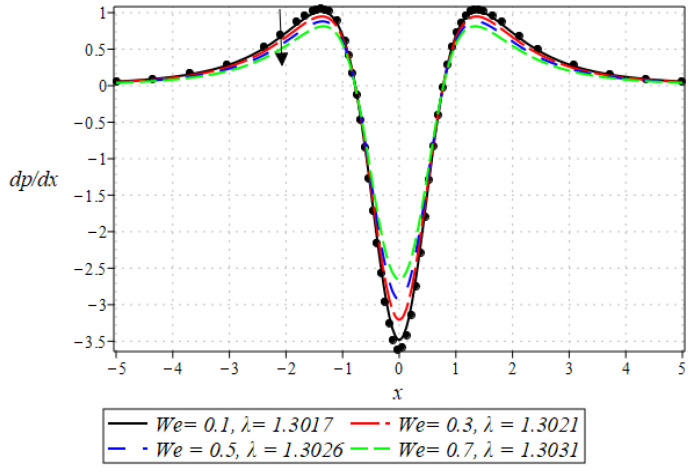
Impact of We on P. G.

**Figure 7:**
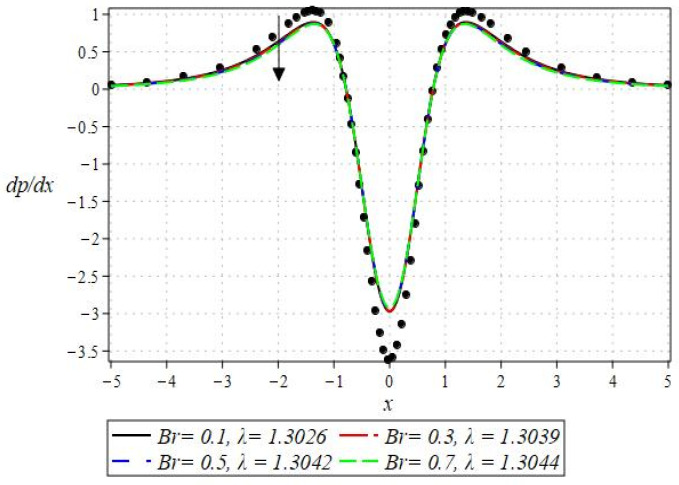
Impact of Br on P. G.

**Figure 8:**
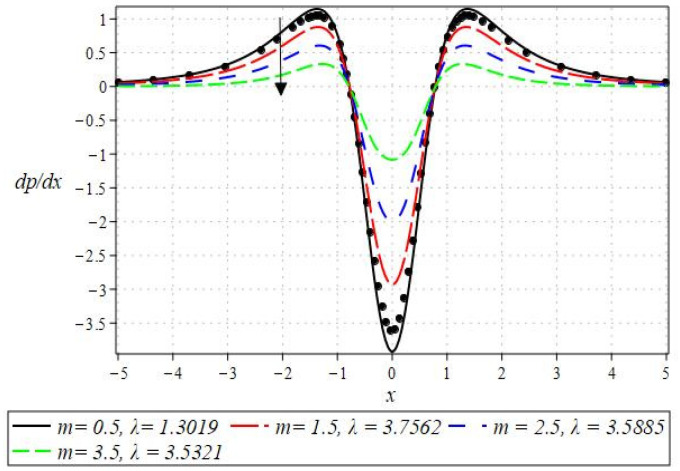
Impact of m on P. G.

**Figure 9:**
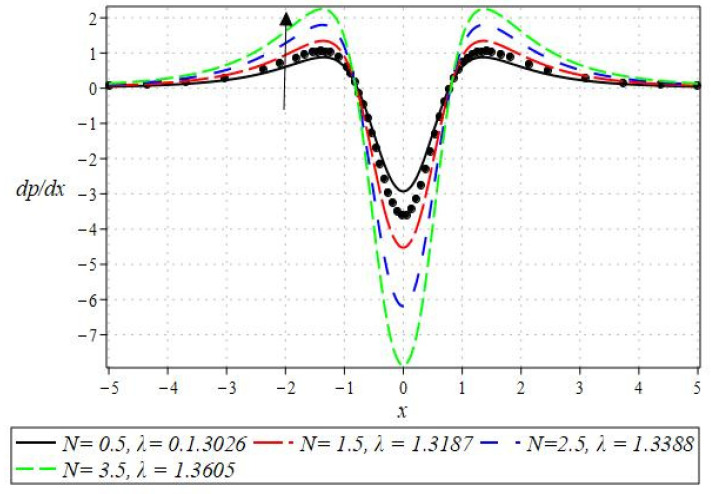
Impact of N on P. G.

**Figure 10:**
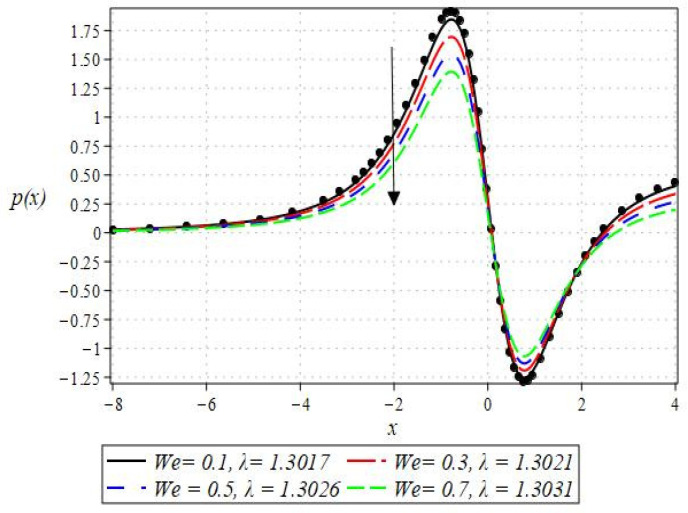
Impact of We on pressure profile.

**Figure 11:**
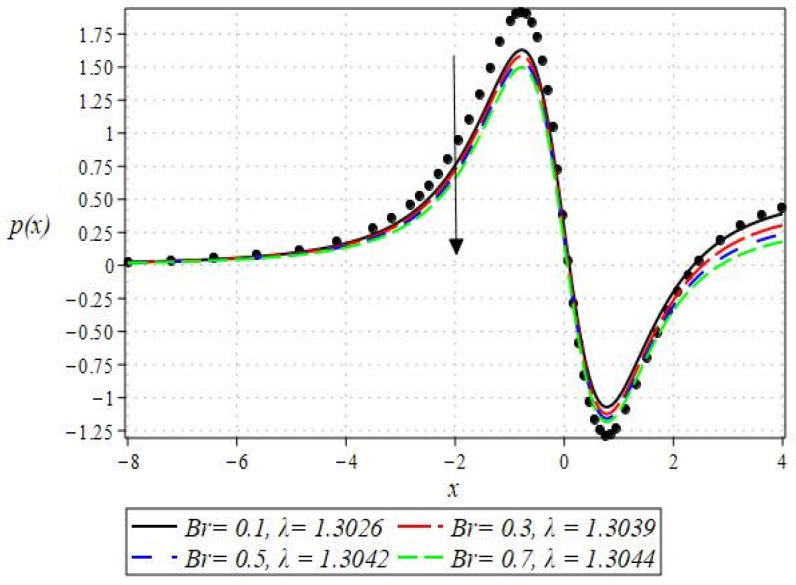
Impact of Br on the pressure profile.

**Figure 12:**
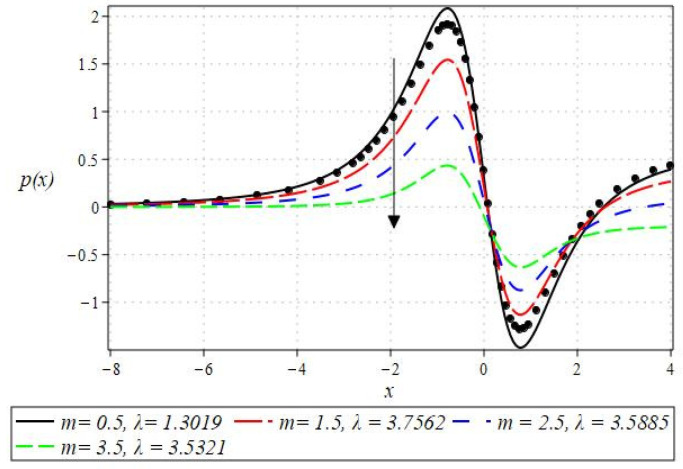
Impact of m on pressure profile.

**Figure 13:**
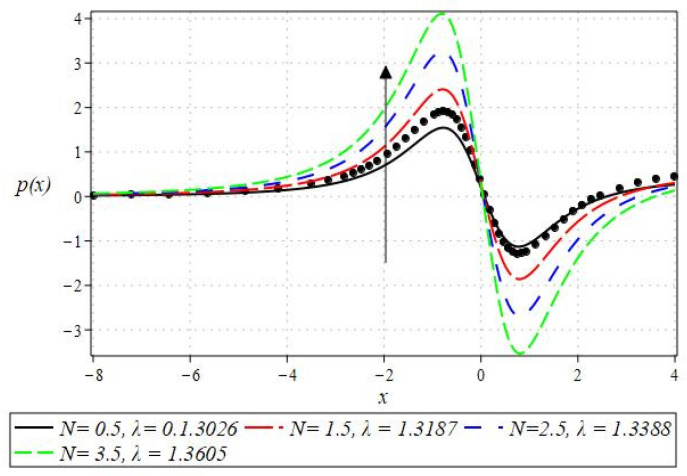
Impact of N on the pressure profile.

The pressure gradient and pressure profile are critical in the roll-over web coating process because they have a direct influence on fluid dynamics and coating quality. The pressure gradient pushes the coating fluid between the rolls and the substrate, ensuring a consistent and controlled flow, which is critical for achieving uniform coating thickness. An appropriate pressure gradient aids in the uniform distribution of the fluid, preventing defects such as streaks, bubbles, and uneven layers. Furthermore, the pressure gradient and pressure profile play important roles in determining the fluid’s shear stress, which influences its deformation and flow behavior. So, the graphical representation of the pressure gradient (P. G.) is depicted in Figure 6, Figure 7, Figure 8 and Figure 9. It has been observed that the pressure gradient decreases with increasing values of We, Br, and m, whereas it increases by increasing the values of N. The pressure profile, which depicts how pressure varies along the coating length, is also important because it influences the stability and uniformity of the coating process. A consistent and well-managed pressure profile ensures that the fluid adheres properly to the substrate, forming a continuous, defect-free coating. Therefore, Figure 10, Figure 11, Figure 12 and Figure 13 are drawn to show how the involved parameters affect the pressure profile versus the dimensionless axial coordinate x and the Newtonian case. From these figures, it is observed that the pressure profile decreases by increasing the values of We, Br and m, whereas increasing by raising the value of N. In addition, the pressure begins to increase from zero, and it reaches its highest levels just before the nip. Physically, it seems true because maximum pressure is required just before the nip to pass the fluid through the channel (nip). 

**Figure 14:**
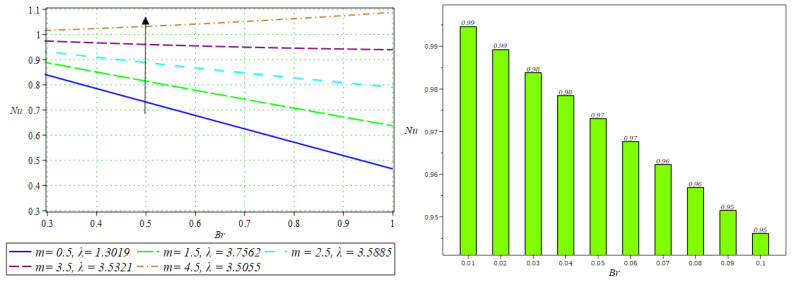
Impact of m on the Nusselt number.

**Figure 15:**
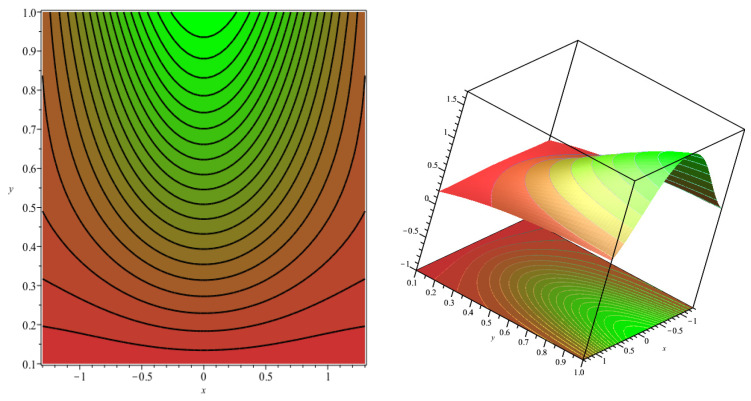
Streamline pattern and surface plots for β=1, m=1.5, N=0.5, We=0.1, Q=1.3015, λ=0.00233, Br=0.5.

Notably, the Newtonian curve for pressure gradient and pressure, which are shown by a solid circle, were obtained for all scenarios when the involved parameters approach zero. These findings for the Newtonian model were previously documented by Greener and Middleman [1]. The validity of our findings is consequently demonstrated by this. Lastly, Figure 14 and Figure 15 were drawn to show the impact of some parameters on the Nusselt number and streamline pattern.

The numerical findings for the volumetric flow rate λ, the exit sheet thickness $H/H_{0}$, the roll separating force, and the power contribution are displayed in Table 1, Table 2 and Table 3 for different values of the We,Br,m, and N. Interestingly, Table 1 demonstrates that as the values of parameter We increase, there is an augmentation in flow rate, coating thickness, the power induced to the material by the rolls, and roll separating force. Similarly, Table 2 exhibits a similar trend of increasing flow rate and coating thickness with increasing Br values, while the magnitude of roll separating force and power input decreases. From Table 3, it has been observed that as m increases from 0.5 to 4.5, there are noticeable changes in these quantities. Interestingly, the power input required to drive the process shows a nonlinear relationship with m. At lower values of m, the power input is negative, suggesting that the system might be operating in a mode where less external energy is needed. However, as m increases beyond 2.5, the power input shifts to positive values and escalates rapidly, reflecting the higher energy required to overcome the increased viscous resistance and maintain the coating process.

## 5. Surface Response Methodology

RSM employs a broad spectrum of mathematical and statistical methodologies to assess the interdependencies between a set of chosen independent parameters (input variables) and corresponding multiple responses/objective functions (output variables). This method is generally applied when multiple input variables influence the responses. Initially, RSM was designed for modeling experimental responses and later extended to model numerical experiments. The distinction lies in the nature of the errors introduced by the responses. In the context of experiments conducted in the physical domain, inaccuracy may be a consequence of measurement errors, whereas in computational simulations, numerical variations from insufficient progress in an iterative process, discretization of continuous dynamic systems, or precision errors. The use of RSM in designing optimization is intended to lessen the cost for complex analysis techniques (e.g., CFD) and numerical-related noises. The specific structure of the correlation between the objective functions and the independent parameters remains unspecified. At the outset of RSM, the key step is to discover a suitable approximation that effectively represents the relationship. In scenarios where a first-order model experiences discrepancies due to variable interactions and complex geometric features, the optimization process can be markedly improved by integrating a second-order model. A standard representation of a second-order paradigm is:

(53) $$ℜ=F_{0}+\sum_{i=1}^{n}F_{i}{℘}_{i}+\sum_{i=1}^{n}F_{ii}{℘}_{i}^{2}+\sum_{i=1}^{n-1}\sum_{j=2}^{n}F_{ij}{℘}_{i}{℘}_{j}+Ε,$$

where regression variables are represented by $F_{0}$, $F_{i}$, $F_{ii}$ and $F_{ij}$, physical factors are ${℘}_{i}$ and ${℘}_{j}$, Ε-residual error, and ℜ-response variables/objective functions. In Table 4, the initial stage outlines the key parameters and their specific ranges.

Following the face-centered CCD technique, the ranges of influenced parameters N, Br, and m are bifurcated into two equal portions. The matrix obtained from the experimental setup is presented in Table 5. An analysis of Table 5 brings to light three distinct point types: factorial, axial, and central with coded values showcased in sections A, B, and C.

To evaluate the precision of the RSM model and the significance of the variable, ANOVA is utilized. It involves essential statistical indicators including *p*-value and F-value. Table 6 and Table 7 provide the respective ANOVA results for shear rate ($\tau_{xy}$), and heat transfer coefficient (Nu). A larger F-value corresponds to a more influential parameter. Similarly, a *p*-value below 0.05 is indicative of statistical significance; otherwise suggests that the terms within the model lack significance. Hence, based on the information presented in Table 6, it is observed that the $AB,A^{2},B^{2}$ surpasses the range of *p*-value and it can be removed from $\tau_{xy}$ the model. Whereas from Table 7, removing $AC,A^{2},$ and $C^{2}$ is necessary to develop a significant Nu model. 

The resulting formulations for the responses $\tau_{xy}$ and Nu can be articulated as below:

(54) $$\tau_{xy}=0.0805+0.0112A-0.0005B+0.0565C+0.0089AC-0.0005BC-0.0063C^{2}$$

(55) $$Nu=0.7964-0.0113A-0.1521B+0.0243C-0.0085AB+0.0185BC+0.0005B^{2}$$

### 5.1. Contour and Surface Plots ($\tau_{xy}$ and Nu)

**Figure 16:**
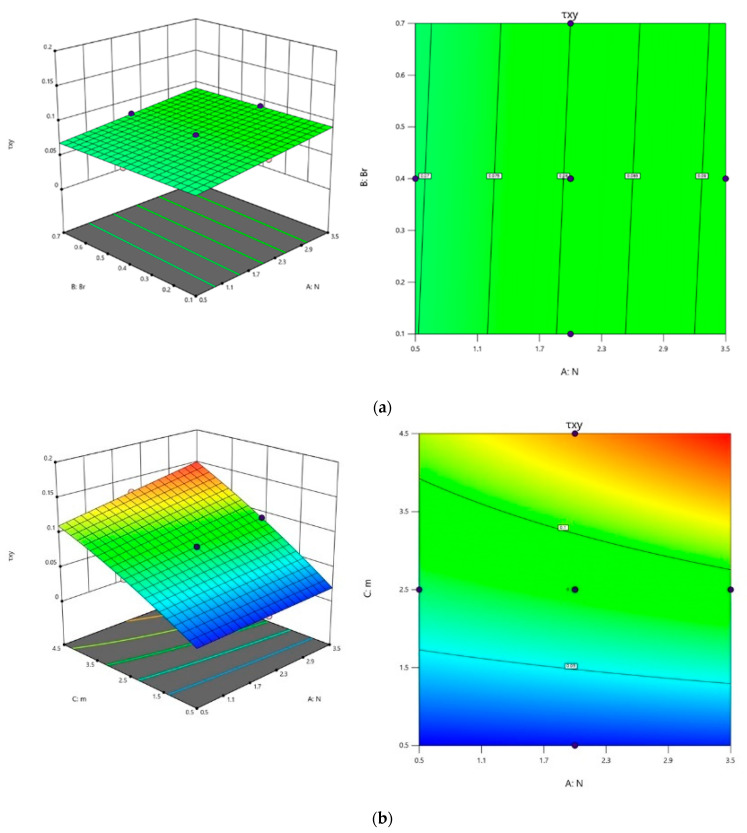

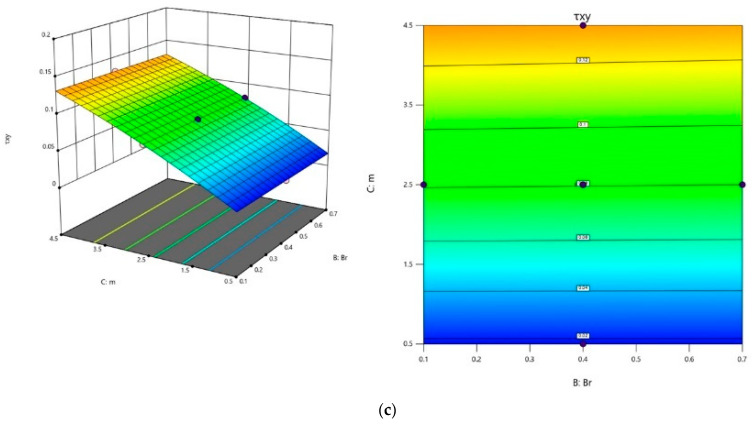
Contour plots and 3D plots for (**a**) N and Br, (**b**) N and m (**c**) Br and m, respectively, and the third variable is maintained at an intermediate level throughout on $\tau_{xy}$.

**Figure 17:**
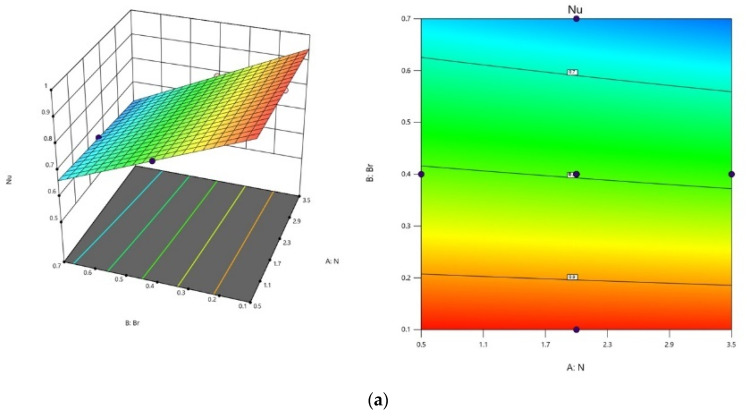

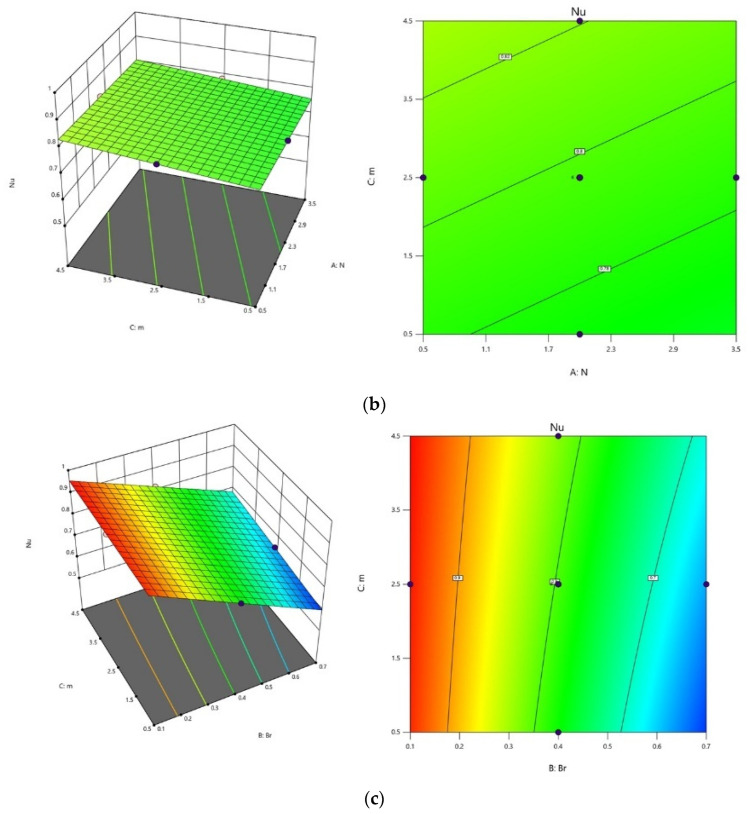
Contour plots and 3D plots for (**a**) N and Br, (**b**) N and m (**c**) Br and m, respectively, and the third variable is maintained at an intermediate level throughout on Nu.

The effect of parameters N, Br, and m on $\tau_{xy}$ and Nu were investigated using surface plots and contour plots in Figure 16 and Figure 17 for Equations (54) and (55). The interconnection among N and Br was analyzed by maintaining the third parameter m as constant. It can be observed from Figure 16a that $\tau_{xy}$ decreases for the lower values of N and higher values of Br. The impact of N and m on $\tau_{xy}$ can be visualized in Figure 16b, i.e., for lower values of N and m, $\tau_{xy}$ decreases, by keeping Br as fixed. Similarly, from Figure 16c it is evident that as m values diminish, and Br values rise, one can observe the minimum value of $\tau_{xy}$.

The visualization in Figure 17a–c elucidates the impact of key factors on Nu. Figure 17a highlights that Nu consistently increases with the lowest N and Br values, while maintaining an intermediate level. The relationship between N, m, and Nu is depicted in Figure 17b. Nu experiences an upward trend with a rise in m, and N is at a low level by fixing Br at a middle level. In Figure 17c, the influence of Br and m is depicted at the intermediate N level. Maintaining m at a high level and Br to a low level yields the maximum Nu value.

### 5.2. Sensitivity Analysis

**Figure 18:**
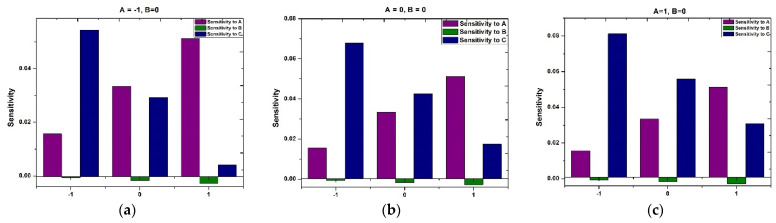
Sensitivity of response variable $\tau_{xy}$ when (**a**) A=-1,B=0, (**b**) A=0,B=0, (**c**) A=1,B=0.

**Figure 19:**
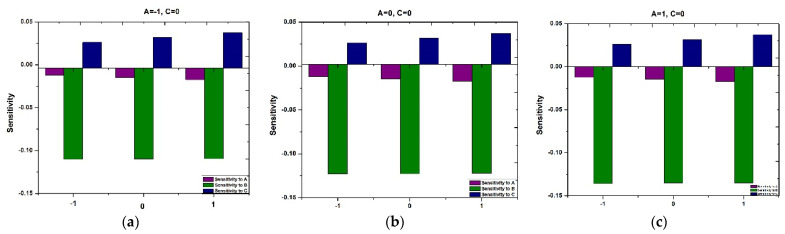
Sensitivity of response variable Nu when (**a**) A=-1,C=0, (**b**) A=0,C=0, (**c**) A=1,C=0.

Examining Figure 18 and Figure 19 reveals the sensitivity patterns of $\tau_{xy}$ and Nu across distinct values of A and C, with B at the middle level and A and B by fixing C at an intermediate level, respectively. Positive and negative bars in the chart denote an enhancement and reduction in $\tau_{xy}$ and Nu. The heights of the bars serve as an indicator of the intensity of the response to the variable.

Investigating the impact of shear stress rate ($\tau_{xy}$) and heat transfer rate (Nu) sensitivity involves employing a quadratic model with coded coefficients, as expressed in Equations (54) and (55). The derivation of sensitivity functions is accomplished by taking partial derivatives of the sensitivity functions are determined by taking partial derivatives of the quadratic model with respect to the coded variables, giving rise to the expression of the following equations.

(56) $$\frac{\partial \tau_{xy}}{\partial A}=0.0112+0.0089C$$

(57) $$\frac{\partial \tau_{xy}}{\partial B}=-0.0005-0.0005C$$

(58) $$\frac{\partial \tau_{xy}}{\partial C}=0.0565+0.0089A-0.0005B-0.0126C$$

(59) $$\frac{\partial Nu}{\partial A}=-0.0113-0.0085B$$

(60) $$\frac{\partial Nu}{\partial B}=-0.1521-0.0085A+0.0185C+0.001B$$

(61) $$\frac{\partial Nu}{\partial C}=0.0243+0.0185B.$$

Figure 18a–c highlight the persistent positivity in sensitivities of $\tau_{xy}$ to A and C across the entire range of C. This observation suggests a continuous upward impact of A and C on $\tau_{xy}$. Meanwhile, the sensitivity of $\tau_{xy}$ to B exhibits negative values, indicating that B has a decreasing effect on $\tau_{xy}$.

Similarly, Figure 19a–c delineate the sensitivity of Nu across various chosen variables. Notably, across all A levels and as B spans from −1  to +1, Nu shows negative sensitivity to A and B and positive sensitivity to C.

### 5.3. Multi-Objective Optimization

**Figure 20:**
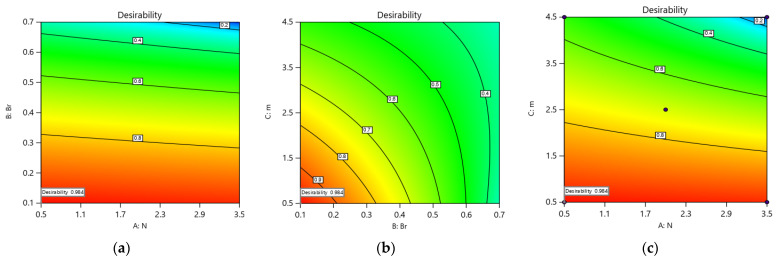
(**a**–**c**): Optimized $\tau_{xy}$ and Nu solution with desirability factors.

RSM was employed for optimization with a focus on multiple criteria. This approach aims to concurrently minimize or maximize one or more objective functions. It finds extensive application across various engineering problems. Here, the goal is to maximize the rate of heat transfer $\left(Nu\right)$, which enhances the efficiency of the model and simultaneously minimizes the shear rate $\left(\tau_{xy}\right)$. The numerical optimization method yields a set of 72 optimal solutions, each with a desirability value ranging from 0 to 1. Optimal solution determination requires identifying the highest desirability, representing the most favorable result. As illustrated in Figure 20a–c, the optimum design is achieved at N=0.500499, Br=0.1, and m=0.501592 resulting in $\tau_{xy}=0.0154145$, Nu=0.945982 and a desirability of 0.984.

## 6. Conclusions

In this paper, the study on the temperature-dependent viscosity analysis of the Powell–Eyring fluid model during a roll-over web coating process sheds light on crucial aspects of non-isothermal roll-coating phenomena. We transformed the dimensional governing momentum and energy equations into dimensionless form, based on the defined dimensionless quantities. By incorporating a variable viscosity model, this research provides valuable insights into optimizing heat transfer efficiency and minimizing shear stress rates. This investigation highlights the significance of considering fluctuating thermophysical characteristics, such as viscosity, in enhancing velocity, pressure, pressure gradient, and temperature distribution profiles during the coating process. Furthermore, the utilization of optimization methodologies, including statistical analysis and response surface methodology, proves essential in maximizing the efficiency of the model and achieving engineering key factors like coating thickness, Nusselt number, and roll transmitted power.

- The velocity profile has a decreasing trend for the increasing values of We and m, whereas the opposite trend was witnessed for increasing values of Br and N.
- A decline in pressure gradient and pressure profile can be noticed by increasing We, Br, and m, whereas opposite behavior can be witnessed for the increasing value of N.
- The streamline plot shows a symmetrical distribution about the vertical axis and indicates a balanced flow on either side of the centerline.
- From sensitivity analysis, it was observed that the sensitivity of $\tau_{xy}$ is positive for A and C. Also, the sensitivity of Nu is positive for C.
- From RSM, it is possible to achieve maximum heat transfer rate and minimum shear stress rate concurrently when N=0.500499,Br=0.1, and m=0.501592 with a desirability of 0.984.
- The Newtonian solution is achieved when the dimensionless parameter becomes zero [1].

Overall, this study contributes to bridging the gap in research concerning the effects of temperature-dependent viscosity on non-Newtonian fluids, particularly the Eyring–Powell fluid, in roll coating applications.

## Figures and Tables

**Table 1:** Impact of we on flow rate, coating thickness, force, and power input.

We	λ	$\frac{H}{H_{0}}$	Separation Force	Power Input
0.1	1.30173	0.650865	−0.09944	−1.86362
0.2	1.30197	0.650985	−0.02874	−2.00728
0.3	1.30220	0.651100	0.07234	−2.15093
0.4	1.30243	0.651215	0.19987	−2.28994
0.5	1.30266	0.651330	0.35074	−2.42104
0.6	1.30290	0.651450	0.52260	−2.54185
0.7	1.30313	0.651565	0.71358	−2.65060
0.8	1.30336	0.651680	0.92231	−2.74596
0.9	1.30360	0.651800	1.14754	−2.82672

**Table 2:** Impact of Br on flow rate, coating thickness, force, and power input.

Br	λ	$\frac{H}{H_{0}}$	Separation Force	Power Input
0.1	1.30266	0.651330	0.35074	−2.42104
0.2	1.30350	0.651750	0.153774	−2.28806
0.3	1.30394	0.651970	0.047609	−2.21690
0.4	1.30415	0.652075	−0.02152	−2.16852
0.5	1.30420	0.652100	−0.07122	−2.13142
0.6	1.30422	0.652110	−0.108580	−2.10079
0.7	1.30435	0.652175	−0.137957	−2.07429
0.8	1.30448	0.652240	−0.161521	−2.05042
0.9	1.30455	0.652275	−0.181215	−2.02833

**Table 3:** Impact of m on flow rate, coating thickness, force, and power input.

m	λ	$\frac{H}{H_{0}}$	Separation Force	Power Input
0.5	1.30196	0.650980	0.390623	−2.82448
1	1.30350	0.651750	0.480159	−2.34299
1.5	1.30362	0.651810	0.668466	−1.71699
2	1.30386	0.651930	0.695152	−0.77848
2.5	1.30404	0.652020	0.546205	0.65600
3	1.30442	0.652210	0.235125	2.79225
3.5	1.30538	0.652690	−0.229888	5.874679
4	1.30640	0.653200	−0.846708	10.20356
4.5	1.30743	0.653715	−1.613521	16.1462

**Table 4:** Three independent selected variables with their respective ranges.

Codes	Variables	Level		
		Low (−1)	Intermediate (0)	High (+1)
A	N	0.5	2	3.5
B	Br	0.1	0.4	0.7
C	m	0.5	2.5	4.5

**Table 5:** Matrix formulation for the tests using the CCD technique.

Std	Run	Space Type	Parameter I	Parameter II	Parameter III	Target I	Target II
			A:N	B:Br	C:m	$\tau_{xy}$	Nu
1	2	Factorial	0.5	0.1	0.5	0.01545	0.9459
2	5	Factorial	3.5	0.1	0.5	0.01997	0.94024
3	17	Factorial	0.5	0.7	0.5	0.01542	0.62173
4	7	Factorial	3.5	0.7	0.5	0.01993	0.58215
5	4	Factorial	0.5	0.1	4.5	0.11156	0.95762
6	16	Factorial	3.5	0.1	4.5	0.15196	0.95196
7	13	Factorial	0.5	0.7	4.5	0.10959	0.70734
8	14	Factorial	3.5	0.7	4.5	0.14968	0.66777
9	6	Axial	0.5	0.4	2.5	0.06925	0.80767
10	3	Axial	3.5	0.4	2.5	0.09176	0.78505
11	11	Axial	2	0.1	2.5	0.08088	0.94893
12	8	Axial	2	0.7	2.5	0.08014	0.64475
13	18	Axial	2	0.4	0.5	0.01769	0.77241
14	15	Axial	2	0.4	4.5	0.13069	0.82031
15	20	Center	2	0.4	2.5	0.08051	0.79636
16	1	Center	2	0.4	2.5	0.08051	0.79636
17	10	Center	2	0.4	2.5	0.08051	0.79636
18	19	Center	2	0.4	2.5	0.08051	0.79636
19	9	Center	2	0.4	2.5	0.08051	0.79636
20	12	Center	2	0.4	2.5	0.08051	0.79636

**Table 6:** Analysis of variance for shear rate ($\tau_{xy}$).

Source	Sum of Squares	df	Mean Square	F-Value	*p*-Value	Coefficient Estimate
Model	0.0340	9	0.0038	5.879 × 10^5^	<0.0001	0.0805
A−N	0.0013	1	0.0013	1.952 × 10^5^	<0.0001	0.0112
B−Br	2.560 × 10^−6^	1	2.560 × 10^−6^	398.17	<0.0001	−0.0005
C−m	0.0319	1	0.0319	4.965 × 10^6^	<0.0001	0.0565
AB	1.280 × 10^−8^	1	1.280 × 10^−8^	1.99	0.1886	0.0000
AC	0.0006	1	0.0006	99,266.00	<0.0001	0.0089
BC	2.184 × 10^−6^	1	2.184 × 10^−6^	339.65	<0.0001	−0.0005
$A^{2}$	5.114 × 10^−12^	1	5.114 × 10^−12^	0.0008	0.9781	−1.364 × 10^−6^
$B^{2}$	3.636 × 10^−11^	1	3.636 × 10^−11^	0.0057	0.9415	3.636 × 10^−6^
$C^{2}$	0.0001	1	0.0001	17,062.06	<0.0001	−0.0063
Residual	6.430 × 10^−8^	10	6.430 × 10^−9^			
Lack of Fit	6.430 × 10^−8^	5	1.286 × 10^−8^			
Pure Error	0.0000	5	0.0000			
Cor Total	0.0340	19				

$R^{2}$ = 1.0000; adjusted $R^{2}$ = 1.0000; predicted $R^{2}$ = 1.0000.

**Table 7:** Analysis of variance for rate of heat transfer (Nu).

Source	Sum of Squares	df	Mean Square	F-Value	*p*-Value	Coefficient Estimate
Model	0.2418	9	0.0269	1.140 × 10^6^	<0.0001	0.7964
A−N	0.0013	1	0.0013	54274.79	<0.0001	−0.0113
B−Br	0.2313	1	0.2313	9.817 × 10^6^	<0.0001	−0.1521
C−m	0.0059	1	0.0059	2.497 × 105	<0.0001	0.0243
AB	0.0006	1	0.0006	24,406.38	<0.0001	−0.0085
AC	1.250 × 10^−11^	1	1.250 × 10^−11^	0.0005	0.9821	1.250 × 10^−6^
BC	0.0027	1	0.0027	1.159 × 105	<0.0001	0.0185
$A^{2}$	5.682 × 10^−13^	1	5.682 × 10^−13^	0.0000	0.9962	−4.545 × 10^−7^
$B^{2}$	6.324 × 10^−7^	1	6.324 × 10^−7^	26.84	0.0004	0.0005
$C^{2}$	5.682 × 10^−13^	1	5.682 × 10^−13^	0.0000	0.9962	−4.545 × 10^−7^
Residual	2.356 × 10^−7^	10	2.356 × 10^−8^			
Lack of Fit	2.356 × 10^−7^	5	4.713 × 10^−8^			
Pure Error	0.0000	5	0.0000			
Cor Total	0.2418	19				

$R^{2}$ = 1.0000; adjusted $R^{2}$ = 1.0000; predicted $R^{2}$ = 1.0000.

## Data Availability

The data presented in this study are available in the paper.

## Nomenclature

U $\left(\frac{m}{s}\right)$	Velocity of the roll
$H_{0}$	Nip region
$\frac{H}{H_{0}}$	Coating thickness
R $\left(m\right)$	Roll radius
ρ $\left(\frac{kg}{m^{3}}\right)$	Fluid density
We	Perturb parameter
m	viscosity variation parameters
λ	Flow rate
$\bar{B}$	The material constant of the Powell–Eyring fluid model
$\bar{C}$	The material constant of the Powell–Eyring fluid model
$\bar{N}$	The material constant of the Powell–Eyring fluid model
ε	Dimensionless number which is square root of ratio of $H_{0}$ to $RH_{0}$
